# Prediction of Drug Targets for Specific Diseases Leveraging Gene Perturbation Data: A Machine Learning Approach

**DOI:** 10.3390/pharmaceutics14020234

**Published:** 2022-01-20

**Authors:** Kai Zhao, Yujia Shi, Hon-Cheong So

**Affiliations:** 1 School of Biomedical Sciences, The Chinese University of Hong Kong, Shatin, Hong Kong SAR, China; zhaokai09@link.cuhk.edu.hk (K.Z.); yj.shi@link.cuhk.edu.hk (Y.S.); 2 KIZ-CUHK Joint Laboratory of Bioresources and Molecular Research of Common Diseases, Kunming Institute of Zoology, Chinese Academy of Sciences, Kunming 650201, China; 3 CUHK Shenzhen Research Institute, Shenzhen 518172, China; 4 Department of Psychiatry, The Chinese University of Hong Kong, Shatin, Hong Kong SAR, China; 5 Margaret K.L. Cheung Research Centre for Management of Parkinsonism, The Chinese University of Hong Kong, Shatin, Hong Kong SAR, China; 6 Brain and Mind Institute, The Chinese University of Hong Kong, Shatin, Hong Kong SAR, China; 7 Hong Kong Branch of the Chinese Academy of Sciences Center for Excellence in Animal Evolution and Genetics, The Chinese University of Hong Kong, Hong Kong SAR, China

**Keywords:** machine learning, drug target, drug repurposing, gene perturbation, expression profiling

## Abstract

Identification of the correct targets is a key element for successful drug development. However, there are limited approaches for predicting drug targets for specific diseases using omics data, and few have leveraged expression profiles from gene perturbations. We present a novel computational approach for drug target discovery based on machine learning (ML) models. ML models are first trained on drug-induced expression profiles with outcomes defined as whether the drug treats the studied disease. The goal is to “learn” the expression patterns associated with treatment. Then, the fitted ML models were applied to expression profiles from gene perturbations (overexpression (OE)/knockdown (KD)). We prioritized targets based on predicted probabilities from the ML model, which reflects treatment potential. The methodology was applied to predict targets for hypertension, diabetes mellitus (DM), rheumatoid arthritis (RA), and schizophrenia (SCZ). We validated our approach by evaluating whether the identified targets may ‘re-discover’ known drug targets from an external database (OpenTargets). Indeed, we found evidence of significant enrichment across all diseases under study. A further literature search revealed that many candidates were supported by previous studies. For example, we predicted PSMB8 inhibition to be associated with the treatment of RA, which was supported by a study showing that PSMB8 inhibitors (PR-957) ameliorated experimental RA in mice. In conclusion, we propose a new ML approach to integrate the expression profiles from drugs and gene perturbations and validated the framework. Our approach is flexible and may provide an independent source of information when prioritizing drug targets.

## 1. Introduction

### 1.1. Background

Traditionally, drug discovery involves a series of steps: target identification, target validation, lead identification, lead optimization, clinical trials, and introduction of the new drug to the market [1]. Nevertheless, the speed of new drug development has been slower than anticipated, despite increasing investment [2]. It is estimated that the cost of developing a new drug is ≈USD 2.6 billion [3]. One of the main reasons for the enormous cost of drug discovery is the high failure rate.

The success of drug development largely depends on the validity of targets. However, most drugs fail to complete the development process due to a lack of efficacy, and this is often due to the wrong target being pursued [4]. Traditionally, drug targets are often identified from hypothesis-driven pre-clinical models, yet pre-clinical models may not always translate well to clinical applications. For some diseases such as psychiatric disorders, current animal or cell models are still far from capturing the complexity of the human disorder [5]. In addition, some have hypothesized that relying on hypothesis-driven studies alone may have led to the ‘filtering’ of findings and publication bias, exacerbating the reliability and reproducibility issues of some research findings [6].

On the other hand, the recent decade has observed a remarkable growth in genomics and other forms of biomedical big data. As increasing amounts of data have been made available, computational methods have attracted increasing attention as they offer a fast, cost-effective, and unbiased way to prioritize promising drug targets. Given the limitation of current approaches and the urgent need to develop therapies for diseases, addressing the problem of target identification and drug development from different angles is essential. We believe that computational and experimental approaches can complement each other to improve the efficiency and reliability of identifying valid drug targets. Given the extremely high cost and time investment in drug development, even if the success rate can be increased by a small margin, the savings (in absolute terms) could be substantial.

### 1.2. Overview of Our Approach

In this study, we present a novel computational target discovery approach based on machine learning (ML) models to expression profiles induced by genetic perturbation. In our approach, ML models are first trained on drug-induced expression profiles, with outcomes defined as whether the drug can treat the studied disease. The goal is to “learn” the expression patterns associated with treatment. Then, the fitted ML models were applied to expression profiles derived from gene perturbations (i.e., overexpression (OE) or knockdown (KD) of specific genes). Afterwards, we could prioritize drug targets based on the predicted probabilities from the ML model, which reflects treatment potential.

Intuitively, for example, the overexpression (OE) of gene *X* leads to an expression profile ‘similar’ to that of five other drugs known to treat diabetes. Then, an agonist targeted at *X* (or other drugs that activate or upregulate *X* and related pathways) may also be useful for treating diabetes. In this case, we expect that the ML model (trained on drugs but applied to gene perturbation data) would output a high predicted probability (of treatment potential) for gene *X*, and it can be prioritized for further studies.

Let us consider an opposite scenario in which the overexpression of gene *Y* increases the disease risk or severity. In this case, we may observe a *lower*-than-expected predicted probability of ‘treatment potential’ from the ML model. Gene *Y* can still be considered a potential drug target for further studies, but here, we expect a *down* regulation of gene *Y* to be associated with disease treatment.

### 1.3. Strengths of Our Approach

Our approach has several potential advantages. Firstly, it provides a general and flexible framework in which any kinds of supervised learning methods can be applied for training. As such, we may leverage the advantages of different, including recently developed, supervised learning algorithms. In addition, our approach is independent of other kinds of evidence usually employed to identify drug targets, for example those used by the OpenTargets platform [7] (e.g., genetic associations, mutation data, expression data, animal models, text mining, etc.). Therefore, the proposed methodology may provide an *independent* source of information when prioritizing targets. In addition, our approach does not rely on the information of known genes or drug targets for a disease; as such, it may be applicable to a wide range of diseases, including disorders with less well-known pathophysiology and targets. The lack of reliance on known disease gene/targets may help discover more novel disease drug targets that are not directly linked to previous ones.

In brief, we first proposed a general framework for identifying drug targets of specific diseases using a machine learning approach, leveraging gene perturbation and drug transcriptome data. Our methodology was applied to several diseases, including type 1 and 2 diabetes mellitus (DM), hypertension (HT), schizophrenia (SCZ), and rheumatoid arthritis (RA). Then, we validated our new framework by assessing its ability to ‘re-discover’ drug targets based on an external established database (OpenTargets). We also found that many candidate targets are supported by the literature and are functionally relevant.

## 2. Methods

We present a general approach for identifying potential drug targets of a specific disease using state-of-the-art ML methods. As described above, ML models were first trained on drug expression profiles to learn the expression patterns associated with treatment of a disease. Then, the trained model was applied to expression profiles after OE or KD to predict the therapeutic potential of up- or downregulation of individual genes.

### 2.1. Datasets

The drug-induced expression profiles and genetically perturbed (OE/KD) expression profiles were downloaded from LINCS (The Library of Integrated Network-Based Cellular Signatures) [8]. For details of the study, please refer to [8]. Briefly, to measure the influence of genetic perturbation on expression, each genetic perturbation (OE/KD) was profiled in triplicate 96 h (h) after application. A single cDNA clone was employed for studies of OE; on the other hand, three distinct shRNAs targeting each gene were profiled for KD experiments. As for expression profiling for drugs, each compound was profiled in triplicate at 6 or 24 h following treatment. Gene expression profiling was based on a reduced representation of the transcriptome (1000 ‘landmark’ genes), which has been shown to produce reliable results compared to standard RNA-seq [8].

The original data at multiple levels of pre-processing are available via the accession GEO: GSE92742 [8]. In the current study, expression data were downloaded from the link (https://github.com/dhimmel/lincs [accessed 26 June 2018]), which provides consensus transcriptional signatures for LINCS L1000 perturbations (see https://think-lab.github.io/d/43/#7 [accessed 26 June 2018]). Briefly, the input signatures were weighted by its Spearman correlation with other input signatures. For consistency, we kept the genes that appeared in both drug-induced and genetically perturbed expression profiles, so ML models trained on drug expression profiles can be directly employed to make predictions on expression data induced from KD/OE experiments. The final drug expression profile dataset consists of 1158 observations, with expression measured in 7467 genes. The dimensions of the OE and KD datasets were 2413 × 7467 and 4326 × 7467, respectively.

### 2.2. Training ML Models on Drug Expression Data to Predict Treatment Potential

The outcome variable (0/1) is defined as whether the drug is indicated for the disease under study. The drug indications were derived from the Anatomical Therapeutic Chemical (ATC) classification system and the MEDication Indication Resource high-precision subset (MEDI-HPS). We employed our proposed approach to predict drug targets for various diseases covering different systems, including hypertension (HT), type 1 and type 2 diabetes mellitus (DM), schizophrenia (SCZ), and rheumatoid arthritis (RA). Indications for HT, DM, and SCZ were extracted from ATC, and indications for RA were extracted from MEDI-HPS, because there is no exact category for RA in ATC. We built prediction models for each disease separately, and four ML classification methods were employed for each disease.

#### 2.2.1. ML Model Building

The model-building procedure largely followed our previous work [9], and we also provide a brief description below. Briefly, we employed four state-of-the-art classification methods, including support vector machine (SVM), gradient boosting machine (GBM), random forest (RF), and logistic regression with the elastic net penalty (EN), to learn the pattern of gene expression profiles associated with treatment of the studied disease [10,11,12,13,14]. As the number of drugs known to treat specific diseases is small, there are few observations with positive outcomes. Following our previous study [9], we performed a weighted analysis by increasing weight of the minority class. SVM, RF, and GBM models were implemented using “scikit-learn” in Python, while EN was implemented with the R package ‘glmnet’. We employed nested 5-fold cross-validation (CV) to choose the optimal hyperparameters and evaluate the performance of corresponding models on hold-out datasets.

Briefly, the dataset was divided into 5 folds using a random seed, and 1/5 of the data (i.e., *N* ≈ 232 observations) was held out for testing in each run. For the remaining 4/5 of observations (*N* ≈ 926), 1/5 of them (*N* ≈ 185) was reserved for tuning hyper-parameters and choosing the best-performing model, while the rest (*N* ≈ 741) was used for training. This is to avoid optimistic bias if one chooses the best hyper-parameters in the test set only. In this study, the test set was only used to evaluate the predictive performance and not involved in hyper-parameter tuning. As a secondary analysis, we also built an ‘ensemble’ model with logistic regression to integrate predicted probabilities from the four ML models for each disease. Drug indication was treated as the outcome, while the predictors were predicted probabilities from the four ML models. We employed a weighted model that up-weighed the minority class, as described above. Please also refer to Supplementary Text for details of model building.

#### 2.2.2. Model Evaluation

Two metrics were used to evaluate the predictive performance of ML models, including area under the receiver operating characteristic curve (ROC-AUC) and area under the precision–recall curve (PR-AUC). PR-AUC may be more instructive in classification performance evaluation when the dataset is imbalanced [15].

### 2.3. External Validation Approach

Validation of drug-disease or drug-target predictions from computational methods has always been a difficult task. As reported by [16], for studies on drug repositioning, a cross-validation approach may overestimate predictive accuracy, as there may be drugs with overlap in the training and testing sets. In addition, highly similar drugs may be split into training and testing sets; hence, the similarity of training and testing sets may be higher than expected than in practice. There may be similar concerns for disease drug target predictions. If one only evaluates the validity of predictions using performance evaluation metrics (e.g., AUC-ROC) under cross-validation alone, this may lead to over-optimistic results. To avoid this problem, we utilized an independent resource to examine whether our approach can ‘re-discover’ known drug targets for diseases from other data sources. Briefly, we validated our results by evaluating whether the identified targets were enriched for those listed by OpenTargets [7], a platform for systematic drug target identification and prioritization. The platform integrates data from genetics, somatic mutations, expression analysis, drugs, animal models, and the literature through robust pipelines and uses an aggregate score to indicate the association of a target with a disease [7].

We applied the models trained on drug expression profiles to OE/KD expression profiles to predict their treatment potentials. Drug targets were downloaded from OpenTargets, with a continuous score (from 0 to 1) indicating the strength of association between the target and disease.

We need to define a cutoff to select relevant genes as ‘valid’ targets for the disease. To avoid arbitrariness in selecting a fixed cutoff, here, we defined a cutoff sequence ranging from 1 to 0 with a step size of 0.2; genes whose association scores are lower than or equal to the cutoff were filtered away, and those genes with association scores higher than the cutoff were considered as “valid” targets. To test for enrichment, we examined whether these ‘valid’ targets from the external database had a higher- or lower-than-expected predicted probability (of treatment potential) from our model when compared to the non-targets. Specifically, we compared the mean predicted probability for genes within the set of ‘valid’ targets against the mean predicted probability of genes not included in the ‘valid’ set. A two-tailed *t*-test was used for this comparison. This method follows closely the principle and methodology of MAGMA [17], which is one of the most widely used programs for gene-based and gene-set analyses in genome-wide associations studies (GWAS). Intuitively, MAGMA compares the mean z-statistic for SNPs/genes within a specified set against those outside the set. (An alternative approach would be to conduct correlation tests between the target score and predicted probabilities; however, the target scores are not normally distributed (with many zeros and ones), which might render such a method unreliable.)

We note that OpenTargets includes the EMBL-EBI Expression Atlas, which contains data for differentially expressed genes in patients against controls for different diseases. However, our drug and gene perturbation expression profiles are based on the L1000 data, and L1000 is not included in the Expression Atlas according to our latest search (as at 3 January 2022). In addition, L1000 data were obtained by the OE/KD of individual genes or application of drugs on primarily cancer cell lines, which do not have a direct relationship with the diseases studied here. We also checked that OpenTargets did not include L1000 as a source for scoring disease–gene associations. Therefore, we believe that OpenTargets represents a reasonably good independent resource for external validation.

## 3. Results

### 3.1. Model Performance

It should be noted that the predictive performance of different ML methods is not the major focus of this study; our main objective is to uncover new disease drug targets and to validate our proposed approach by testing for its ability to ‘re-discover’ known targets.

The average predictive performance of different ML models, measured in AUC-ROC and AUC-PR, is presented in Table S1. In terms of AUC-ROC, SVM performed the best for SCZ, while EN performed the best for DM and RA. GBM slightly outperformed others for HT. In terms of AUC-PR, SVM performed the best in DM and SCZ datasets, but GBM and EN showed the best performance for HT and RA, respectively. Note that AUC-PR is dependent on the proportion of positive outcome; hence, the low AUC-PR observed is expected given the relatively small number of drugs indicated for each disorder. Table S2 briefly summarizes the overall number of drugs included for each disease.

We also carried out further analysis to evaluate the correlation of predicted probabilities from different models for each disease under study (Table S3). We found significant positive correlations between the predicted probabilities. However, most of the correlations were moderate, suggesting that different models can still produce different predictions.

### 3.2. External Validation

The results of enrichment test for ‘known’ drug targets from OpenTargets (based on OE data) are shown in Table 1, Table 2, Table 3, Table 4 and Table 5. Overall, for drug targets identified from OE data, we observed significant enrichment (with FDR (false discovery rate) adjusted *p*-values < 0.05) for at least one ML method and score threshold for all the diseases under study (Table 1, Table 2, Table 3, Table 4 and Table 5).

For DM and HT, we observed significant enrichment across multiple thresholds and most of the ML methods with FDR < 0.05, indicating that the proposed method indeed ‘re-discovered’ known targets more than expected by chance. For RA, significant enrichment was mainly observed for prediction models based on RF or GBM. For SCZ and bipolar disorder (BP), which shared anti-psychotics as treatment, the enrichment was not as strong, but suggestive enrichment (FDR < 0.1) was observed especially for targets based on SVM for SCZ and EN for BP.

As a test of the robustness of our results to different random seeds, we also picked two diseases (HT/RA) and repeated the analysis using a different random seed for sample splitting. The results were broadly similar with significant enrichment observed (see Tables S4 and S5).

On the other hand, apart from a few significant findings for HT, no statistically significant enrichment was observed for targets identified from KD data. The results are shown in supplementary tables (Table S6).

### 3.3. Literature Support of Potential Targets

In order to validate the functional relevance of our identified potential targets, we conducted a literature search of the 10 targets with the highest and lowest predicted probabilities for each disease (please see Table S7 for a list of these targets) based on targets identified from OE data. These highlighted targets were selected from a total of 2413 genes subject to OE experiments. As described in the introduction, for targets with high predicted probabilities, we expect that upregulation of the gene may be associated with therapeutic potential; for targets with lower-than-expected predicted probabilities, we predict that downregulation of the gene may be associated with therapeutic potential.

Selected targets with literature support are discussed below and highlighted in Table 6. Note that our proposed approach does not utilize any prior knowledge of disease–gene associations.

#### 3.3.1. Schizophrenia/Bipolar Disorder

Schizophrenia and bipolar disorder share similar clinical characteristics, and antipsychotics are indicated for both disorders. The two disorders are also highly genetically correlated [18]. Therefore, we tested for target enrichment for both SCZ and BP based on our model trained on the ATC-SCZ dataset. Our study suggests that the overexpression of *DRD1* may be associated with treatment effects on SCZ. It was reported that insufficient D1 receptor signaling was associated with cognitive deficits and that working memory deficits may be relieved by treatments that augment D1 receptor stimulation, indicating that drugs acting on this potential target may restore cognitive dysfunction in SCZ [19]. Indeed, DRD1 agonist has been tested in a clinical trial for cognitive enhancement [20]. Moderate improvement was observed on some cognitive tests, including the CogState battery and attention domain of the MATRICS cognitive battery, although no significant improvement was detected for working memory. Other studies also suggested a role of DRD1 in the pathophysiology of SCZ [21]. Taken together, DRD1 may be a potential therapeutic target for SCZ.

HIF1AN, another target identified in our models, has been proven to suppress HIF1A’s transcriptional ability, which thus can affect HIF1A’s ability in regulating hypoxia-inducible genes [22,23]. Hypoxia may be involved in the pathogenesis of SCZ. For example, a methylome-wide association study (MWAS) of SCZ identified many top hits related to hypoxia [24]. *HIF1A* is also proposed as a candidate gene for SCZ, considering the association between *HIF1A* and intrinsic hypoxia occurring in the developing brain that may lead to complex changes in neurodevelopment [25,26]. HIF1A may enhance vascular growth (hence reducing hypoxia) via controlling the expression of vascular endothelial growth factor (VEGF) [27]. We found that the inhibition of *HIF1AN* expression may be associated with the therapeutical effect on SCZ, which is in line with the direction of effect from the above studies.

A few other targets may also be associated with SCZ, as supported by other studies. For example, ADCY9 is involved in glutamate and GABA neurotransmission pathways [28], and damaging *de novo* mutations have been identified in the gene [29]. Another potential target, *RPA2*, showed differential expression in a study of pluripotent stem cell-derived neurons from SCZ patients [30]. As for another candidate target, RhoA, a study showed reduced mRNA expression of *RhoA* in dorsolateral prefrontal cortex of SCZ subjects compared to controls [31]. NDUFS4 is another target we identified. Interestingly, a GWAS study revealed that an SNP (rs67017972) close to *NDUFS4* was significantly associated with verbal memory in SCZ patients [32].

#### 3.3.2. Rheumatoid Arthritis

A number of selected potential targets such as SMAD7, TGFBR2, FGFR10P, and PSMB8 are supported by previous studies. It was reported that *SMAD7* expression was largely reduced in synovial tissues of RA patients, and mouse models also showed that SMAD7 deficiency increased risk to autoimmune arthritis [33]. In addition, it was shown that the intra-articular overexpression of *SMAD7* relieved experimental arthritis [34]. These results support our prediction that the overexpression of *SMAD7* may improve RA.

Regarding another potential target, TGFBR2, it has been reported that TGFBR2 plays an important role in chondrogenesis [35]. Current results showed that up to 40% of RA patients are resistant to methotrexate, which is the first-line therapy for RA. Peres et al. reported that the drug resistance of methotrexate was linked to a reduction of CD39 expression due to the impairment in TGF-β signaling, and TGF-β increases CD39 expression on regulatory T cells (Tregs) via the activation of TGFBR2 [36]. The authors also observed that patients non-responsive to methotrexate had reduced expression of *TGFBR2* in Tregs compared to responsive patients. In this connection, the overexpression of *TGFBR2* may reverse the impairment of TGF-β signaling, which is consistent with our prediction that *TGFBR2* overexpression may be useful for RA treatment. In addition, hypermethylation of TGFBR2 (associated with decreased expression) was found in RA samples [37]. Taken together, the results above indicate that *TGFBR2* expression levels might be linked to RA disease activity.

Additionally, FGFR10P was identified as a possible target for RA by our study. An LD block on chromosome 6 (6q27) that contains the genes *CCR6* and *FGFR10P* was observed to be associated with increased risks for several autoimmune diseases, such as RA, Crohn’s disease, and vitiligo [38,39,40,41,42,43].

Moreover, our model predicted that the inhibition of PSMB8 may induce treatment effects on RA. This is directly supported by experimental evidence from animal studies. It was observed that treatment with a PSMB8 inhibitor (PR-957) can ameliorate experimental RA in mice [44]. The drug led to a decrease in cellular infiltration, cytokine production, and autoantibodies in the RA mouse model. In a similar vein, several studies also reported that PSMB8 inhibitors reversed autoreactive immune responses and showed therapeutic effects in animal models of autoimmune encephalomyelitis, colitis, and Hashimoto’s thyroiditis [45,46,47]. Regarding evidence from human genetics studies, an SNP in the *PSMB8* (*LMP7*) gene was also found to be associated with juvenile RA [48].

Our study also predicted inhibition of IL-21 receptor (IL-21R) as a potential treatment for RA. An animal study reported that IL-21 receptor expression on B cells contributed to the development of collagen-induced arthritis (CIA) [49]. This implies that IL-21R inhibitors may hinder the development of CIA. The role of IL-21R in RA is also supported by other studies. Berberine (BBR) is a drug that has been suggested as a potential treatment for RA [50], and a recent study [51] showed that BBR inhibits IL-21/IL-21R-dependent autophagy, leading to a reduction of proliferation of arthritic fibroblast-like synoviocytes, which in turn may lead to the amelioration of RA.

We also predicted that the inhibition of LTβR (lymphotoxin β receptor) may be associated with therapeutic effects. Interestingly, a phase-1 randomized controlled trial of the safety and efficacy of pateclizumab (a drug that inhibits LTα1β2-LTβR interactions) showed that the drug was well tolerated and demonstrated preliminary evidence of clinical activity compared to placebo [52]. In a subsequent phase II study, pateclizumab resulted in a higher response rate than placebo treatment at 12 weeks, but the difference was not statistically significant [53]. However, CXCL13 (a biomarker of RA activity) [54] serum levels decreased significantly after pateclizumab treatment.

Another potential target identified was DAXX. It was reported that the accumulation of DAXX in promyelocytic leukemia (PML) protein nuclear bodies (NBs) may promote inflammatory disorders, which is in line with our prediction that the downregulation of this target may be beneficial to treatment [55].

#### 3.3.3. Diabetes Mellitus

Previous studies also support several potential targets, and our study suggested that the overexpression of these targets may be associated with treatment effects on DM. First, Mayumi et al. found that mutations of *NR0B2*, also known as *SHP*, was associated with type 2 DM in a Japanese sample. It was reported that the mutant proteins show significantly reduced activities [56]. Another study showed that the inhibitory effect of metformin (one of the most commonly used drugs for DM) on hepatic gluconeogenesis may be mediated through the expression of NR0B2 [57].

We also identified Fos as a potential target for DM (with OE favoring treatment). It was found that insulin could induce *c-Fos* mRNA expression in neurons [58], fibroblasts [59], and pancreatic beta-cells [60]. Another study showed that *c-Fos* upregulation increased beta-cell proliferation, insulin secretion, and cellular survival, which is mediated by the activation of Nkx6.1. On the other hand, *c-Fos* knockdown inhibits Nkx6.1-mediated beta-cell proliferation and reduces insulin secretion [61].

QPRT (quinolinate phosphoribosyltransferase) was also found to be a potential target for DM. It is an enzyme involved in the kynurenine pathway, which may be involved in diabetes pathogenesis [62]. In a recent clinical study, the expression of *QPRT* in the subcutaneous compartment was negatively correlated with HbA1c, fasting glycemia, and 120 min glycemia [62]. This is consistent with our finding that the upregulation of this target may be associated with therapeutic potential.

We also revealed MAGED1 as a potential drug target, and predicted that the upregulation of the gene may be associated with the treatment of DM. One study concluded that MAGED1-deficient mice showed hyperphagia and reduced motor activity, which led to the development of obesity [63]. Another subsequent animal study [64] observed a similar phenomenon in which MAGED1-deficient mice showed late-onset obesity, owing to reduced energy expenditure and physical activities. The study also found that *MAGED1* expression was reduced during adipogenesis, and loss of MAGED1 led to increased pre-adipocyte proliferation and differentiation in vitro. MAGED1 also reduced the stability and transcriptional activity of PPAR-gamma, which is the target for thiazolidinediones (a class of anti-diabetic medication).

Several other identified targets were also shown in previous studies to be associated with DM. For example, *GADD45A* was suggested as a diabetes-associated gene, which might be involved in both diabetic cardiomyopathy and DM-induced baroreflex dysfunction [65]. Regarding another potential target, *TSPAN8*, a SNP in this gene was associated with insulin release and sensitivity in a genetic association study [66]. Another target of interest was PPP2R1A, which encodes a constant regulatory subunit of protein phosphatase 2 (PP2A). It was found that the podocyte-specific loss of PP2A worsened diabetic glomerulopathy and accelerated the progression of diabetic kidney disease [67]. In addition, PPP2R1A was discovered to interact with IRS1 (Insulin receptor substrate 1), which is a key mediator of insulin signal transduction implicated in Type 2 DM [68].

We also identified TANK-binding kinase 1 (TBK1) as a potential target. The inhibition of TBK1 to treat DM is supported by a few previous studies. It has been suggested that a combination of immunomodulators and agents that specifically increase the mass of functional β-cells favors the treatment of type 1 DM [69]. In a study using a zebrafish model of type 1 DM, Xu et al. revealed that the inhibition of TBK1/IKKε (IκB kinase ε) led to β-cell regeneration [70]. Another study [71] showed that TBK1 is expressed mainly in beta cells of mammalian islets and that the genetic silencing of TBK1 led to an elevated expression of genes and proteins that are important for beta cell proliferation. On the other hand, TBK1 expression in beta cells was raised in human type 2 DM islets.

Our study also suggested that USF1 and HLA-DMB are potential targets for DM. Risk alleles of *USF1* have been found to be associated with cardiovascular disease and type 2 DM risk in a number of studies [72,73,74]. Moreover, another study showed that a risk allele within *USF1* appeared to remove the inductive effect of insulin on *USF1* expression, which in turn affected the expression of other target genes, contributing to increased risk of cardiometabolic diseases [75]. *HLA-DMB* was also reported to be associated with type 1 DM in several human genetic studies [76,77,78].

#### 3.3.4. Hypertension

Our models identified TCF7L2 as a potential target for hypertension. *TCF7L2* is a well-established susceptibility gene for type 2 DM [79], and given the high comorbidity rate and possibly shared pathophysiology between DM and hypertension [80], further studies into this target may be warranted. In addition, a study of the Thai elderly population suggested that the SNP rs290487 in *TCF7L2* may contribute to risks of hypertension regardless of type 2 DM [81]. Another cohort study concluded that both a parental history of diabetes and the *TCF7L2* at-risk variant were associated with a higher incidence of hypertension after controlling for other cardiometabolic risk factors [82].

Considering another potential target, ATP5A1, a pharmacological network analysis of Compound Uncaria Hypotensive Tablet (a Chinese medication for hypertension) revealed that the therapeutic effect of this drug may be associated with actions on ATP synthetases, including ATP5A1 [83]. Another study revealed that the expression of *ATP5A1* was significantly decreased in spontaneously hypertensive rats compared with controls [84]. These results may further support our finding that the overexpression of *ATP5A1* may be associated with therapeutic effects.

Another target of interest is FADD, which is also a marker of apoptosis and apoptosis that may be implicated in atherosclerosis [85]. Cohort studies reported that a high plasma level of FADD was associated with increased incidence of coronary events and ischemic stroke [86,87]. Considering the strong associations between hypertension, stroke, and coronary heart diseases [88], the findings from these studies are supportive of the role of FADD and our prediction that the inhibition of FADD expression may be associated with treatment of hypertension (or its complications).

Interestingly, TSPAN8, which was suggested by our approach as a DM drug target, was also identified as a potential target for hypertension. As argued above, given the comorbidities and possibly shared metabolic pathways [80], TSPAN8 may also be an interesting candidate.

Another potential target we found was NFE2L2 (also known as Nrf2), which was directly supported by an animal study. Bai et al. [89] showed that the injection of tert-butylhydroquinone (t-BHQ), a selective Nrf2 activator, significantly reduced mean arterial pressure, plasma norepinephrine levels, and sympathetic nerve activities in hypertensive rats. tBHQ also reduced levels of reactive oxygen species and decreased inflammatory cytokine release in the periventricular nucleus (PVN). In addition, the knockdown of Nrf2 in the PVN abrogated the therapeutic effects of tBHQ on HT. Another study also suggested a role of Nrf2 activation in endothelial protection and the attenuation of oxidative stress [90]. These findings are consistent with our model prediction that the activation or overexpression of NFE2L2 may be associated with therapeutic benefits.

Some other potential targets, such as DUSP6 and HOXB13, are also supported by the literature. Zoe et al. [91] found that genes from the DUSP family may contribute to hypertensive heart disease; specifically, for *DUSP6*, its expression was upregulated in spontaneously hypertensive rats compared to controls. The above results were consistent with our finding that the inhibition of *DUSP6* may have a protective treatment effect. For HOXB13, it was reported that the knockdown of *HOXB13* can reduce the cytotoxicity caused by various oxidative stress inducers [92,93], and an increasing number of studies suggest that oxidative stress has a key role in the pathogenesis of hypertension [94]. As for another target, ETV1, in a study of human left atrium (LA) samples, ETV1 was downregulated in cardiac pressure overload, which may in turn be associated with both electrical and structural remodeling [95].

## 4. Discussion

### 4.1. Overview

In this study, we presented a novel ML-based computational approach to identify promising drug targets. To our knowledge, this work is the first to employ ML methods to leverage both drug-induced and genetically perturbed expression data to discover potential drug targets for specific diseases.

Our approach is general as it can incorporate any supervised learning algorithms. To validate our method, we examined whether it may ‘re-discover’ known targets based on other sources of data. Indeed, we observed that top genes from our models were enriched for targets from the OpenTargets platform. Encouragingly, a number of targets highlighted by our proposed method were also supported by the literature.

### 4.2. Relevant Works

We highlight a few relevant works on drug target prediction here. Kandoi et al. reviewed machine learning and system biology applications in distinguishing drug targets from non-targets [96]. Several studies explored the biological properties of known drug targets by ML methods to predict the druggability of proteins [97,98,99,100,101]. For example, Kumari et al. proposed a sequence-based prediction model and leveraged information such as amino acid composition and amino acid property group composition to predict whether a new target may be druggable. They also performed a comprehensive comparison of several ML methods [100]. In another study, eight key properties of human drug targets were extracted and learned by SVM to discover new targets [97,98,99,100,101]. A similar study extracted simple physicochemical properties from known drug targets to predict targets against non-targets [101].

Regarding network-based approaches, Costa et al. [102] leveraged interaction network topological features together with tissue expression and subcellular localization data to predict druggable genes. In another work, Li et al. employed the topological features of a protein–protein interaction network to identify potential drug targets [99]. Emig et al. presented an integrated network-based method to predict drug targets based on disease gene expression profiles and an interaction network, and some novel drug targets for scleroderma and cancer were reported [103].

However, our study is different from the previous studies in several aspects. One of the most important differences is that the focus of most of the above studies (except [103]) is to predict *in general* whether a protein may serve as a drug target (i.e., distinguishing targets from non-targets). They did not address the problem of predicting whether a protein is a target *for a specific disease,* such as diabetes or SCZ. As discussed above, network-based methods are useful and have been proposed for uncovering disease drug targets. However, they are relatively dependent on similarity between entities and known drug targets; hence, they may be less capable of discovering novel targets. In addition, network-based approaches usually require good knowledge of gene–gene (or protein–protein) and disease–gene interactions. It may not be easy to define such interactions accurately, and different sources may suggest different patterns of interactions. Therefore, the edges may need to be defined arbitrarily.

There are relatively few studies that employed gene perturbation data to predict drug targets, but a recent study [104] has leveraged such data to identify tentative targets. The authors proposed pairwise learning and joint learning methods constructed on chemically and genetically perturbed gene expression profiles to predict the targets of different chemicals [104]. They also constructed a drug–protein–disease network for drug repurposing. However, the methodologies and objectives of our study and ref [104] are different. We proposed ML methods to assess how the expression profiles from gene perturbations are related to those of drugs. Ref [104] mainly employed Pearson correlation and linear models to assess the similarity between transcriptomic changes from gene perturbation and those from drugs. An advantage of our approach is that by employing ML methods (e.g., SVM, random forests, boosted trees), we may accommodate complex non-linear relationships and interactions between features. The study [104] used transcriptomic data from gene perturbations mainly to predict drug–protein interactions; prediction of disease-specific drug targets was performed in a separate analysis using networks (which requires knowledge of the known therapeutic targets of studied diseases). As discussed above, network-based methods have their own limitations. We proposed an alternative new approach, which integrates transcriptomic data with ML approaches in a unified framework to predict drug targets *for specific diseases.*

One of our previous works has employed an ML approach for drug repositioning, leveraging drug expression data [18]. However, the objectives are different from the current study, in which we aim to uncover novel drug targets. In practice, drug repositioning may not always be feasible (for example, due to side effects of existing drugs), and there are also important hurdles to drug repositioning efforts, such as patent considerations, regulatory barriers, and organizational hurdles in industry, as reviewed by Pushpakom et al. [105]. As a result, revealing new targets remains a very important goal in drug development and pharmaceutical research. Unlike our previous work, here, we have covered diseases other than psychiatric disorders. In addition, gene perturbation data have not been used in the previous study.

There seems to be some discrepancies in the performance of enrichment tests across diseases, with DM and HT having the most significant enrichment. We are uncertain about the exact reasons, but there are many possible factors that may lead to such differences. For example, it is unknown how well the experiments on cell lines can capture the actual expression changes in human tissues relevant to the studied diseases. The L1000 gene perturbation data are primarily based on several cancer cell lines, which may not correlate very well with actual expression changes in relevant tissues, e.g., the brain for our study on SCZ. Another speculation is that the range of mechanisms underlying HT and DM drugs may be slightly more diverse than RA or SCZ, which may facilitate the ML models to learn expression patterns conducive to therapeutic effects. For example, most drugs for SCZ are based on similar mechanisms related to dopaminergic blockade.

We also could not conclude with certainty why some ML approaches may perform worse in some cases. It is possible that some methods may not be able to capture very well the expression patterns (which can differ across diseases) contributing to therapeutic potential, especially in view of the imbalanced datasets. This may in turn affect the performance in the target enrichment tests.

### 4.3. Strengths and Limitations

As described earlier, there are important strengths of our approach. Our approach is general and highly flexible, can incorporate any supervised ML methods, is independent of other sources of evidence commonly employed to identify drug targets, and does not rely on knowledge on known disease genes/targets. However, there are also several limitations. One limitation is that our ML prediction model-building datasets are highly imbalanced, as only a small number of drugs are usually indicated for each disease. In order to address this issue, we increased the class weight of the minority group. There are other strategies to address issues, such as SMOTE (Synthetic Minority Oversampling Technique) [106], but whether strategies such as SMOTE can address this issue in high-dimensional settings is still unclear and will be a topic for further investigations. Our primary objective of this study is to present a general framework for prioritizing drug targets leveraging drug expression and gene perturbation data; as such, we have not investigated in great depths ways to improve the ML model itself, which will be left as a topic for further work. For example, it may be worthwhile to explore other kinds of ML approaches and ways of combining predictions from different models; the models may also be further evaluated by repeated CV or bootstrap approaches.

Another aspect is that we observed significant enrichment for the identified targets primarily in the OE datasets but not in KD datasets. One hypothesis is that some off-target effects may interfere with the expression profiles in KD experiments, leading to greater difficulties in finding relevant drug targets [8]. How to overcome or reduce the influence of off-target effects remains an area for further studies. Here, we have employed enrichment tests to examine ‘re-discovery’ of known drug targets from other sources of data and showed that many targets may be clinically/biologically relevant based on the literature. Nevertheless, further experimental and clinical studies are required to confirm our findings. In addition, further works are required to elucidate the mechanisms underlying the drugs that may act on the identified targets.

## 5. Conclusions

This study presents a general computational framework to prioritize drug targets for various diseases. Under the framework, different kinds of ML methods can be utilized. We applied four ML methods to identify potential drug targets of four disorders. External validation showed that the top candidates are enriched for targets selected by independent lines of evidence from a large external database (OpenTargets). We also found that previous studies provided support to a number of targets identified by our approach.

Finding promising targets for diseases is crucial to drug development. However, it is impractical to perform in-depth experimental studies on every possible target for each disease. Computational methods offer a cheap, fast, and systematic high-throughput approach to guide the prioritization of targets. We hope our presented framework will provide an additional way to prioritize drug targets, which may benefit future drug development.

## Figures and Tables

**Table 1:** Enrichment test of the predicted targets for HT (enrichment for targets listed in OpenTargets).

Threshold	SVM	RF	GBM	EN
	*p*-Value	*p*-Value	*p*-Value	*p*-Value
1	**4.81 × 10^−^** **^3^**	**3.31 × 10^−2^**	9.81 × 10^−2^	**2.09 × 10^−2^**
0.8	**4.32 × 10^−3^**	**2.56 × 10^−2^**	8.29 × 10^−2^	**1.61 × 10^−2^**
0.6	**4.41 × 10^−4^**	**4.94 × 10^−3^**	1.76 × 10^−2^	**5.68 × 10^−3^**
0.4	**8.26 × 10^−4^**	**4.86 × 10^−3^**	1.60 × 10^−2^	**1.12 × 10^−2^**
0.2	**2.04 × 10^−3^**	**1.19 × 10^−2^**	2.91 × 10^−2^	**2.17 × 10^−2^**
0	1.56 × 10^−1^	6.84 × 10^−1^	1.96 × 10^−1^	1.12 × 10^−1^

Enrichment *p*-values are shown. Four machine learning methods were used to train a model on expression data to predict treatment potential, and the model was fitted to expression profiles after gene perturbation. The false discovery rate (FDR) approach was employed to correct for multiple testing. *p*-values with corresponding FDR < 0.05 are in bold, while *p*-values with corresponding FDR between 0.05 and 0.1 are in italics. The first column is the threshold of the ‘relevance’ score (available from OpenTargets) above which we defined a gene as a drug ‘target’. Enrichment was tested against the targets listed in the OpenTargets database, which is the same as below. SVM: support vector machines; EN: logistic regression with elastic net regularization; RF: random forest; GBM, gradient boosted machines. For Table 1, Table 2, Table 3, Table 4 and Table 5, the predicted targets are based on expression data from overexpression (OE) experiments. The results from KD data are listed in Table S6.

**Table 2:** Enrichment test of the predicted targets for DM.

Threshold	SVM	RF	GBM	EN
	*p*-Value	*p*-Value	*p*-Value	*p*-Value
1	5.78 × 10^−2^	6.53 × 10^−2^	**3.28 × 10^−5^**	**2.32 × 10^−3^**
0.8	**2.33 × 10^−2^**	2.13 × 10^−2^	**4.31 × 10^−5^**	**1.67 × 10^−3^**
0.6	**1.26 × 10^−2^**	1.37 × 10^−2^	**1.61 × 10^−5^**	**1.57 × 10^−3^**
0.4	**2.32 × 10^−2^**	4.32 × 10^−2^	**1.72 × 10^−3^**	**5.22 × 10^−3^**
0.2	6.43 × 10^−1^	3.67 × 10^−1^	**1.71 × 10^−2^**	**3.55 × 10^−2^**
0	3.00 × 10^−1^	6.24 × 10^−1^	8.10 × 10^−1^	8.21 × 10^−2^

**Table 3:** Enrichment test of the predicted targets for RA.

Threshold	SVM	RF	GBM	EN
	*p*-Value	*p*-Value	*p*-Value	*p*-Value
1	1.18 × 10^−1^	**6.23 × 10^−4^**	**2.01 × 10^−2^**	9.22 × 10^−1^
0.8	1.36 × 10^−1^	**3.93 × 10^−4^**	**8.53 × 10^−3^**	9.94 × 10^−1^
0.6	1.18 × 10^−1^	1.41 × 10^−1^	3.67 × 10^−1^	9.20 × 10^−1^
0.4	6.44 × 10^−1^	**1.14 × 10^−2^**	**2.25 × 10^−2^**	2.69 × 10^−1^
0.2	3.12 × 10^−1^	8.47 × 10^−2^	4.15 × 10^−2^	8.48 × 10^−2^
0	3.71 × 10^−1^	7.01 × 10^−1^	1.96 × 10^−1^	2.56 × 10^−1^

**Table 4:** Enrichment test of the predicted targets for SCZ (for targets of SCZ listed in OpenTargets).

Threshold	SVM	RF	GBM	EN
	*p*-Value	*p*-Value	*p*-Value	*p*-Value
1	3.32 × 10^−1^	2.84 × 10^−1^	2.57 × 10^−1^	3.56 × 10^−1^
0.8	4.66 × 10^−1^	2.47 × 10^−1^	2.64 × 10^−1^	2.80 × 10^−1^
0.6	2.18 × 10^−2^	7.78 × 10^−1^	9.94 × 10^−1^	7.47 × 10^−1^
0.4	1.91 × 10^−2^	8.62 × 10^−1^	7.97 × 10^−1^	3.84 × 10^−1^
0.2	7.00 × 10^−2^	8.97 × 10^−1^	5.42 × 10^−1^	7.18 × 10^−1^
0	7.11 × 10^−1^	1.85 × 10^−1^	9.01 × 10^−1^	3.14 × 10^−1^

**Table 5:** Enrichment test of the predicted targets for SCZ (for targets of bipolar disorder listed in OpenTargets).

Threshold	SVM	RF	GBM	EN
	*p*-Value	*p*-Value	*p*-Value	*p*-Value
1	4.14 × 10^−1^	7.24 × 10^−1^	5.88 × 10^−1^	5.59 × 10^−1^
0.8	4.66 × 10^−1^	7.58 × 10^−1^	9.43 × 10^−1^	9.43 × 10^−1^
0.6	8.57 × 10^−1^	6.84 × 10^−1^	2.56 × 10^−1^	2.90 × 10^−2^
0.4	8.57 × 10^−1^	6.84 × 10^−1^	2.56 × 10^−1^	2.90 × 10^−2^
0.2	4.13 × 10^−1^	3.17 × 10^−1^	6.03 × 10^−2^	**1.45 × 10^−3^**
0	1.31 × 10^−2^	7.97 × 10^−1^	1.91 × 10^−1^	4.76 × 10^−1^

**Table 6:** Literature support of selected drug target candidates.

Potential Target	Disease	Direction of Expression Associated with Treatment Effect (as Predicted)	Literature Support/Functional Relevance
DRD1	SCZ	up	Insufficient D1 receptor signaling may be associated with cognitive deficits; D1 agonist has been tested in a clinical trial for cognitive symptoms in SCZ, with moderate improvement in some cognitive tasks observed
HIF1AN	SCZ	down	Hypoxia may play a role in SCZ by affecting neurodevelopment; genetic studies showed that HIFs may be involved in SCZ
ADCY9	SCZ	up	Involved in glutamate and GABA neurotransmission; *de novo* mutation in the gene may be associated with SCZ
NDUFS4	SCZ	down	An SNP close to *NDUFS4* was significantly associated with verbal memory in SCZ in a GWAS
SMAD7	RA	up	*Smad7* expression reduced in synovial tissues of RA patients; mouse models showed that Smad7 deficiency increased risk to autoimmune arthritis; intra-articular overexpression of *Smad7* relieved experimental arthritis
TGFBR2	RA	up	Linked to resistance of methotrexate treatment and non-responsive patient had reduced expression of the gene in regulatory T cells; hypermethylation (associated with decreased expression) found in RA samples
FGFR10P	RA	down	An LD block containing this gene was found in the GWAS of RA and other autoimmune conditions
PSMB8	RA	down	Directly supported by experimental evidence from animal studies: treatment with a PSMB8 inhibitor (PR-957) ameliorated experimental RA in mice.
IL-21R	RA	down	IL-21 receptor expression on B cells contributed to collagen-induced arthritis in animal studies; berberine inhibits IL-21/IL-21R-dependent autophagy and has been suggested as a treatment for RA
LTBR	RA	down	A phase-1 RCT showed that pateclizumab (a drug that inhibits LTα1β2–LTβR interactions) led to a reduction in RA clinical activity compared to placebo, although no statistically significant difference was shown in a phase 2 trial
NR0B2	DM	up	Mutations (associated with reduced activities) in the gene associated with DM; inhibitory effect of metformin on hepatic gluconeogenesis may be mediated through expression of *NR0B2*
Fos	DM	up	Insulin induced *c-Fos* mRNA expression in various cell types including beta-cells; c-Fos upregulation increased beta-cell proliferation, insulin secretion, and cellular survival
QPRT	DM	up	Expression of *QPRT* in subcutaneous compartment negatively correlated with HbA1c, fasting glycemia, and 120 min glycemia in a clinical study
MAGED1	DM	up	MAGED1-deficient mice showed hyperphagia and reduced motor activity, which is associated with obesity (shown by two animal studies). *MAGED1* expression was reduced during adipogenesis, and loss of MAGED1 led to increased pre-adipocyte proliferation and differentiation *in vitro*
PPP2R1A	DM	up	Encodes a regulatory subunit of PP2A; podocyte-specific loss of PP2A worsened diabetic glomerulopathy and accelerated the progression of diabetic kidney disease; interacts with IRS1 (Insulin receptor substrate 1), which is a key mediator of insulin signal transduction implicated in Type 2 DM
TBK1	DM	down	TBK1 is expressed primarily in beta cells of mammalian islets; inhibition of TBK1/IKKε (IκB kinase ε) led to increased β-cell regeneration
TCF7L2	HT	up	A well-established susceptibility gene for DM found in GWAS (DM and HT are highly comorbid and may share common pathways); genetic association studies showed associations of SNPs in *TCF7L2* with HT
ATP5A1	HT	up	Reduced expression in HT rats; network analysis showed that actions of a Chinese drug on HT may be mediated through this target
FADD	HT	down	Cohort studies reported that a high plasma level of FADD was associated with increased incidence of coronary events and ischemic stroke
NFE2L2	HT	up	A selective Nrf2 activator (tBHQ) significantly reduced mean arterial pressure, plasma norepinephrine levels, and sympathetic nerve activities in hypertensive rats; tBHQ also reduced levels of reactive oxygen species and decreased inflammatory cytokine release in the periventricular nucleus (PVN)

RCT, randomized controlled trial.; tBHQ, tert-butylhydroquinone.

## Data Availability

Full data of LINCS L1000 perturbational profiles can be accessed via the GEO accession code: GSE70138. Consensus transcriptional signatures for LINCS L1000 perturbations are available at https://github.com/dhimmel/lincs. ATC drug categories can be found at https://www.genome.jp/brite/br08303, and the drug indications from MEDI-HPS can be downloaded from https://www.vumc.org/cpm/cpm-blog/medi-ensemble-medication-indication-resource-0. The OpenTargets database is available at https://platform.opentargets.org/ (accessed on 30 November 2021).

## Supplementary Materials

The following are available online at https://www.mdpi.com/article/10.3390/pharmaceutics14020234/s1. Supplementary Text: Hyperparameter tuning and weighted analysis, Figure S1: Receiver-operating curves (ROC) of different machine learning methods across four datasets, Table S1: Average predictive performance of different machine learning methods across four datasets, Table S2: Summary of the number of drugs in nested cross validation in our study, Table S3: Pearson correlation of predicted probabilities from different ML models for each disease, Table S4: Average predictive performance of different machine learning methods across two datasets, Table S5: Enrichment test of the predicted targets for HT and RA, Table S6: Enrichment test results from Knockdown (KD) data (including 5 sub-tables), Table S7: List of identified targets (the 10 targets with the highest and lowest predicted probabilities of treatment potential are shown; including four sub-tables showing targets for each disease).

## References

[1] Chen Y.-P.P., Chen F. (2008). Identifying targets for drug discovery using bioinformatics. Expert Opin. Ther. Targets.

[2] Pammolli F., Magazzini L., Riccaboni M. (2011). The productivity crisis in pharmaceutical R&D. Nat. Rev. Drug Discov..

[3] DiMasi J.A., Grabowski H.G., Hansen R.W. (2016). Innovation in the pharmaceutical industry: New estimates of R&D costs. J. Health Econ..

[4] Shih H., Zhang X., Aronov A.M. (2018). Drug discovery effectiveness from the standpoint of therapeutic mechanisms and indications. Nat. Rev. Drug Discov..

[5] Nestler E.J., Hyman S.E. (2010). Animal models of neuropsychiatric disorders. Nat. Neurosci..

[6] Schneider G. (2018). Automating drug discovery. Nat. Rev. Drug Discov..

[7] Koscielny G., An P., Carvalho-Silva D., Cham J., Fumis L., Gasparyan R., Hasan S., Karamanis N., Maguire M., Papa E. (2016). Open Targets: A platform for therapeutic target identification and validation. Nucleic Acids Res..

[8] Subramanian A., Narayan R., Corsello S.M., Peck D.D., Natoli T.E., Lu X., Gould J., Davis J.F., Tubelli A.A., Asiedu J.K. (2017). A next generation connectivity map: L1000 platform and the first 1,000,000 profiles. Cell.

[9] Zhao K., So H.-C. (2018). Drug Repositioning for Schizophrenia and Depression/Anxiety Disorders: A Machine Learning Approach Leveraging Expression Data. IEEE J. Biomed. Health Inform..

[10] Freund Y., Schapire R.E. (1997). A Decision-Theoretic Generalization of On-Line Learning and an Application to Boosting. J. Comput. Syst. Sci..

[11] Friedman J.H. (2001). Greedy function approximation: A gradient boosting machine. Ann. Stat..

[12] Breiman L. (2001). Random forests. Mach. Learn..

[13] Zou H., Hastie T. (2005). Regularization and variable selection via the elastic net. J. R. Stat. Soc. Ser. B (Stat. Methodol.).

[14] Cortes C., Vapnik V. (1995). Support-vector networks. Mach. Learn..

[15] Davis J., Goadrich M. The relationship between precision-recall and ROC curves. Proceedings of the 23rd International Conference on Machine Learning.

[16] Guney E. Reproducible Drug Repurposing: When Similarity Does Not Suffice. Proceedings of the Pacific Symposium on Biocomputing 2017.

[17] de Leeuw C.A., Mooij J.M., Heskes T., Posthuma D. (2015). MAGMA: Generalized gene-set analysis of GWAS data. PLoS Comput. Biol..

[18] Cardno A.G., Owen M.J. (2014). Genetic relationships between schizophrenia, bipolar disorder, and schizoaffective disorder. Schizophr. Bull..

[19] Goldman-Rakic P.S., Castner S.A., Svensson T.H., Siever L.J., Williams G.V. (2004). Targeting the dopamine D1 receptor in schizophrenia: Insights for cognitive dysfunction. Psychopharmacologia.

[20] Girgis R.R., Van Snellenberg J.X., Glass A., Kegeles L.S., Thompson J.L., Wall M., Cho R.Y., Carter C.S., Slifstein M., Abi-Dargham A. (2016). A proof-of-concept, randomized controlled trial of DAR-0100A, a dopamine-1 receptor agonist, for cognitive enhancement in schizophrenia. J. Psychopharmacol..

[21] Kaalund S.S., Newburn E.N., Ye T., Tao R., Li C., Deep-Soboslay A., Herman M.M., Hyde T.M., Weinberger D.R., Lipska B.K. (2014). Contrasting changes in DRD1 and DRD2 splice variant expression in schizophrenia and affective disorders, and associations with SNPs in postmortem brain. Mol. Psychiatry.

[22] Hu C.J., Wang L.Y., Chodosh L.A., Keith B., Simon M.C. (2003). Differential roles of hypoxia-inducible factor 1α (HIF-1α) and HIF-2α in hypoxic gene regulation. Mol. Cell. Biol..

[23] Mahon P.C., Hirota K., Semenza G.L. (2001). FIH-1: A novel protein that interacts with HIF-1α and VHL to mediate repression of HIF-1 transcriptional activity. Genes Dev..

[24] Aberg K.A., McClay J.L., Nerella S., Clark S., Kumar G., Chen W., Khachane A.N., Xie L., Hudson A., Gao G. (2014). Methylome-wide association study of schizophrenia: Identifying blood biomarker signatures of environmental insults. JAMA Psychiatry.

[25] Schmidt-Kastner R., van Os J., Steinbusch H.W., Schmitz C. (2006). Gene regulation by hypoxia and the neurodevelopmental origin of schizophrenia. Schizophr. Res..

[26] Schmidt-Kastner R., Guloksuz S., Kietzmann T., Van Os J., Rutten B.P.F. (2020). Analysis of GWAS-Derived Schizophrenia Genes for Links to Ischemia-Hypoxia Response of the Brain. Front. Psychiatry.

[27] Maltepe E., Simon M.C. (1998). Oxygen, genes, and development: An analysis of the role of hypoxic gene regulation during murine vascular development. J. Mol. Med..

[28] Martin C., Jacobi J.S., Nava G., Jeziorski M.C., Clapp C., De La Escalera G.M. (2007). GABA Inhibition of Cyclic AMP Production in Immortalized GnRH Neurons Is Mediated by Calcineurin-Dependent Dephosphorylation of Adenylyl Cyclase 9. Neuroendocrinology.

[29] Gulsuner S., Walsh T., Watts A.C., Lee M.K., Thornton A.M., Casadei S., Rippey C., Shahin H., Nimgaonkar V.L., Go R.C. (2013). Spatial and Temporal Mapping of De Novo Mutations in Schizophrenia to a Fetal Prefrontal Cortical Network. Cell.

[30] Roussos P., Guennewig B., Kaczorowski D.C., Barry G., Brennand K. (2016). Activity-Dependent Changes in Gene Expression in Schizophrenia Human-Induced Pluripotent Stem Cell Neurons. JAMA Psychiatry.

[31] Hill J.J., Hashimoto T., A Lewis D. (2006). Molecular mechanisms contributing to dendritic spine alterations in the prefrontal cortex of subjects with schizophrenia. Mol. Psychiatry.

[32] Nakahara S., Medland S., Turner J.A., Calhoun V.D., Lim K., Mueller B.A., Bustillo J.R., O’Leary D., Vaidya J., McEwen S. (2018). Polygenic risk score, genome-wide association, and gene set analyses of cognitive domain deficits in schizophrenia. Schizophr. Res..

[33] Zhou G., Sun X., Qin Q., Lv J., Cai Y., Wang M., Mu R., Lan H.-Y., Wang Q.-W. (2018). Loss of Smad7 Promotes Inflammation in Rheumatoid Arthritis. Front. Immunol..

[34] Chen S.-Y., Shiau A.-L., Wu C.-L., Wang C.-R. (2016). Intraarticular overexpression of Smad7 ameliorates experimental arthritis. Sci. Rep..

[35] Zhong N., Sun J., Min Z., Zhao W., Zhang R., Wang W., Tian J., Tian L., Ma J., Li D. (2012). MicroRNA-337 is associated with chondrogenesis through regulating TGFBR2 expression. Osteoarthr. Cartil..

[36] Peres R.S., Donate P.B., Talbot J., Cecilio N.T., Lobo P.R., Machado C.C., de Lima K.A., Oliveira R.D., Carregaro V., Nakaya H. (2018). TGF-β signalling defect is linked to low CD39 expression on regulatory T cells and methotrexate resistance in rheumatoid arthritis. J. Autoimmun..

[37] Nakano K., Whitaker J.W., Boyle D.L., Wang W., Firestein G.S. (2012). DNA methylome signature in rheumatoid arthritis. Ann. Rheum. Dis..

[38] Yi X., Du L., Hou S., Li F., Chen Y., Kijlstra A., Yang P. (2013). FGFR1OP tagSNP but Not CCR6 Polymorphisms Are Associated with Vogt-Koyanagi-Harada Syndrome in Chinese Han. PLoS ONE.

[39] Chu X., Pan C.M., Zhao S.X., Liang J., Gao G.Q., Zhang X.M., Yuan G.Y., Li C.G., Xue L.Q., Shen M. (2011). A genome-wide association study identifies two new risk loci for Graves’ disease. Nat. Genet..

[40] Stahl E.A., Raychaudhuri S., Remmers E.F., Xie G., Eyre S., Thomson B.P., Li Y., Kurreeman F.A.S., Zhernakova A., Hinks A. (2010). Genome-wide association study meta-analysis identifies seven new rheumatoid arthritis risk loci. Nat. Genet..

[41] Kochi Y., Okada Y., Suzuki A., Ikari K., Terao C., Takahashi A., Yamazaki K., Hosono N., Myouzen K., Tsunoda T. (2010). A regulatory variant in CCR6 is associated with rheumatoid arthritis susceptibility. Nat. Genet..

[42] Barrett J.C., Hansoul S., Nicolae D.L., Cho J.H., Duerr R.H., Rioux J.D., Brant S.R., Silverberg M.S., Taylor K.D., Barmada M.M. (2008). Genome-wide association defines more than 30 distinct susceptibility loci for Crohn’s disease. Nat. Genet..

[43] Quan C., Ren Y.-Q., Xiang L.-H., Sun L.-D., Xu A.-E., Gao X.-H., Chen H.-D., Pu X.-M., Wu R.-N., Liang C.-Z. (2010). Genome-wide association study for vitiligo identifies susceptibility loci at 6q27 and the MHC. Nat. Genet..

[44] Muchamuel T., Basler M., Aujay M.A., Suzuki E., Kalim K.W., Lauer C., Sylvain C., Ring E.R., Shields J., Jiang J. (2009). A selective inhibitor of the immunoproteasome subunit LMP7 blocks cytokine production and attenuates progression of experimental arthritis. Nat. Med..

[45] Nagayama Y., Nakahara M., Shimamura M., Horie I., Arima K., Abiru N. (2012). Prophylactic and therapeutic efficacies of a selective inhibitor of the immunoproteasome for Hashimoto’s thyroiditis, but not for Graves’ hyperthyroidism, in mice. Clin. Exp. Immunol..

[46] Basler M., Mundt S., Muchamuel T., Moll C., Jiang J., Groettrup M., Kirk C.J. (2014). Inhibition of the immunoproteasome ameliorates experimental autoimmune encephalomyelitis. EMBO Mol. Med..

[47] Basler M., Dajee M., Moll C., Groettrup M., Kirk C.J. (2010). Prevention of Experimental Colitis by a Selective Inhibitor of the Immunoproteasome. J. Immunol..

[48] Prahalad S., Kingsbury D.J., A Griffin T., Cooper B.L., Glass D.N., Maksymowych W.P., A Colbert R. (2001). Polymorphism in the MHC-encoded LMP7 gene: Association with JRA without functional significance for immunoproteasome assembly. J. Rheumatol..

[49] Sakuraba K., Oyamada A., Fujimura K., Spolski R., Iwamoto Y., Leonard W.J., Yoshikai Y., Yamada H. (2016). Interleukin-21 signaling in B cells, but not in T cells, is indispensable for the development of collagen-induced arthritis in mice. Arthritis Res..

[50] Wang X.-H., Jiang S.-M., Sun Q.-W. (2011). Effects of berberine on human rheumatoid arthritis fibroblast-like synoviocytes. Exp. Biol. Med..

[51] Dinesh P., Rasool M. (2019). Berberine mitigates IL-21/IL-21R mediated autophagic influx in fibroblast-like synoviocytes and regulates Th17/Treg imbalance in rheumatoid arthritis. Apoptosis.

[52] Emu B., Luca D., Offutt C., Grogan J.L., Rojkovich B., Williams M.B., Tang M.T., Xiao J., Lee J.H., Davis J.C. (2012). Safety, pharmacokinetics, and biologic activity of pateclizumab, a novel monoclonal antibody targeting lymphotoxin α: Results of a phase I randomized, placebo-controlled trial. Arthritis Res. Ther..

[53] Kennedy W.P., Simon J.A., Offutt C., Horn P., Herman A., Townsend M.J., Tang M.T., Grogan J.L., Hsieh F., Davis J.C. (2014). Efficacy and safety of pateclizumab (anti-lymphotoxin-α) compared to adalimumab in rheumatoid arthritis: A head-to-head phase 2 randomized controlled study (The ALTARA Study). Arthritis Res. Ther..

[54] Bechman K., Dalrymple A., Southey-Bassols C., Cope A.P., Galloway J.B. (2020). A systematic review of CXCL13 as a biomarker of disease and treatment response in rheumatoid arthritis. BMC Rheumatol..

[55] Meinecke I., Cinski A., Baier A., Peters M.A., Dankbar B., Wille A., Drynda A., Mendoza H., Gay R.E., Hay R.T. (2007). Modification of nuclear PML protein by SUMO-1 regulates Fas-induced apoptosis in rheumatoid arthritis synovial fibroblasts. Proc. Natl. Acad. Sci. USA.

[56] Enya M., Horikawa Y., Kuroda E., Yonemaru K., Tonooka N., Tomura H., Oda N., Yokoi N., Yamagata K., Shihara N. (2008). Mutations in the small heterodimer partner gene increase morbidity risk in Japanese type 2 diabetes patients. Hum. Mutat..

[57] Kim Y.D., Park K.-G., Lee Y.-S., Park Y.-Y., Kim D.-K., Nedumaran B., Jang W.G., Cho W.-J., Ha J., Lee I.-K. (2008). Metformin Inhibits Hepatic Gluconeogenesis through AMP-Activated Protein Kinase–Dependent Regulation of the Orphan Nuclear Receptor SHP. Diabetes.

[58] Heidenreich K.A., Zeppelin T., Robinson L.J. (1993). Insulin and insulin-like growth factor I induce c-fos expression in postmitotic neurons by a protein kinase C-dependent pathway. J. Biol. Chem..

[59] Stumpo D.J., Blackshear P. (1986). Insulin and growth factor effects on c-fos expression in normal and protein kinase C-deficient 3T3-L1 fibroblasts and adipocytes. Proc. Natl. Acad. Sci. USA.

[60] Uhles S., Moede T., Leibiger B., Berggren P.-O., Leibiger I.B. (2007). Selective gene activation by spatial segregation of insulin receptor B signaling. FASEB J..

[61] Ray J.D., Kener K.B., Bitner B.F., Wright B.J., Ballard M.S., Barrett E.J., Hill J.T., Moss L.G., Tessem J.S. (2016). Nkx6. 1-mediated insulin secretion and β-cell proliferation is dependent on upregulation of c-Fos. FEBS Lett..

[62] Favennec M., Hennart B., Caiazzo R., Leloire A., Yengo L., Verbanck M., Arredouani A., Marre M., Pigeyre M., Bessede A. (2015). The kynurenine pathway is activated in human obesity and shifted toward kynurenine monooxygenase activation. Obesity.

[63] Dombret C., Nguyen T., Schakman O., Michaud J.L., Hardin-Pouzet H., Bertrand M., De Backer O. (2012). Loss of Maged1 results in obesity, deficits of social interactions, impaired sexual behavior and severe alteration of mature oxytocin production in the hypothalamus. Hum. Mol. Genet..

[64] Wang Q., Tang J., Jiang S., Huang Z., Song A., Hou S., Gao X., Ruan H.-B. (2018). Inhibition of PPARγ, adipogenesis and insulin sensitivity by MAGED1. J. Endocrinol..

[65] Wang N., Yang C., Xie F., Sun L., Su X., Wang Y., Wei R., Zhang R., Li X., Yang B. (2012). Gadd45α: A Novel Diabetes-Associated Gene Potentially Linking Diabetic Cardiomyopathy and Baroreflex Dysfunction. PLoS ONE.

[66] Grarup N., Andersen G., Krarup N.T., Albrechtsen A., Schmitz O., Jørgensen T., Borch-Johnsen K., Hansen T., Pedersen O. (2008). Association testing of novel type 2 diabetes risk alleles in the JAZF1, CDC123/CAMK1D, TSPAN8, THADA, ADAMTS9, and NOTCH2 loci with insulin release, insulin sensitivity, and obesity in a population-based sample of 4,516 glucose-tolerant middle-aged Danes. Diabetes.

[67] Zhong Y., Lee K., Deng Y., Ma Y., Chen Y., Li X., Wei C., Yang S., Wang T., Wong N.J. (2019). Arctigenin attenuates diabetic kidney disease through the activation of PP2A in podocytes. Nat. Commun..

[68] Caruso M., Ma D., Msallaty Z., Lewis M., Seyoum B., Al-Janabi W., Diamond M., Abou-Samra A.B., Højlund K., Tagett R. (2014). Increased Interaction with Insulin Receptor Substrate 1, a Novel Abnormality in Insulin Resistance and Type 2 Diabetes. Diabetes.

[69] Pozzilli P., Maddaloni E., Buzzetti R. (2015). Combination immunotherapies for type 1 diabetes mellitus. Nat. Rev. Endocrinol..

[70] Xu J., Jia Y.F., Tapadar S., Weaver J.D., Raji I.O., Pithadia D.J., Javeed N., García A.J., Choi D.S., Matveyenko A.V. (2018). Inhibition of TBK1/IKKε promotes regeneration of pancreatic β-cells. Sci. Rep..

[71] Jia Y.-F., Jeeva S., Xu J., Heppelmann C.J., Jang J.S., Slama M.Q., Tapadar S., Oyelere A.K., Kang S.-M., Matveyenko A.V. (2020). TBK1 regulates regeneration of pancreatic β-cells. Sci. Rep..

[72] Komulainen K., Alanne M., Auro K., Kilpikari R., Pajukanta P., Saarela J., Ellonen P., Salminen K., Kulathinal S., Kuulasmaa K. (2006). Risk alleles of USF1 gene predict cardiovascular disease of women in two prospective studies. PLoS Genet..

[73] Meex S.J., van Vliet-Ostaptchouk J.V., van der Kallen C.J., van Greevenbroek M.M., Schalkwijk C.G., Feskens E.J., Blaak E.E., Wijmenga C., Hofker M.H., Stehouwer C.D. (2008). Upstream transcription factor 1 (USF1) in risk of type 2 diabetes: Association study in 2000 Dutch Caucasians. Mol. Genet. Metab..

[74] Holzapfel C., Baumert J., Grallert H., Müller A.M., Thorand B., Khuseyinova N., Herder C., Meisinger C., Hauner H., E Wichmann H. (2008). Genetic variants in the USF1 gene are associated with low-density lipoprotein cholesterol levels and incident type 2 diabetes mellitus in women: Results from the MONICA/KORA Augsburg case–cohort study, 1984–2002. Eur. J. Endocrinol..

[75] Naukkarinen J., Nilsson E., Koistinen H.A., Soderlund S., Lyssenko V., Vaag A., Poulsen P., Groop L., Taskinen M.R., Peltonen L. (2009). Functional variant disrupts insulin induction of USF1: Mechanism for USF1-associated dyslipidemias. Circ. Cardiovasc. Genet..

[76] Sang Y.M., Yan C., Zhu C., Ni G.C., Hu Y.M. (2005). Relationship between HLA-DMA, DMB Alleles and Type 1 Diabetes in Chinese. HK J. Paediatr. (New Ser.).

[77] Siegmund T., Donner H., Braun J., Usadel K., Badenhoop K. (1999). HLA-DMA and HLA-DMB alleles in German patients with type 1 diabetes mellitus. Tissue Antigens.

[78] Kim S.-S., Hudgins A.D., Yang J., Zhu Y., Tu Z., Rosenfeld M.G., DiLorenzo T.P., Suh Y. (2021). A comprehensive integrated post-GWAS analysis of Type 1 diabetes reveals enhancer-based immune dysregulation. PLoS ONE.

[79] Grant S.F. (2019). The TCF7L2 Locus: A Genetic Window Into the Pathogenesis of Type 1 and Type 2 Diabetes. Diabetes Care.

[80] Cheung B.M., Li C. (2012). Diabetes and hypertension: Is there a common metabolic pathway?. Curr. Atheroscler. Rep..

[81] Rattanatham R., Settasatian N., Settasatian C., Decharatchakul N., Sarutipaiboon I., Kanyalert S., Boonpalit N. (2017). Genetic polymorphism in TCF7L2 and risk of hypertension in Thai elderly subjects. Atherosclerosis.

[82] Bonnet F., Roussel R., Natali A., Cauchi S., Petrie J., Laville M., Yengo L., Froguel P., Lange C., Lantieri O. (2013). Parental history of type 2 diabetes, TCF7L2 variant and lower insulin secretion are associated with incident hypertension. Data from the DESIR and RISC cohorts. Diabetologia.

[83] Long H.-P., Lin X.-Y., Wang Y.-H., Ren W.-Q., Shao L., Zhang W., Tan Y.-S. (2018). Explore mechanism of Compound Uncaria Hypotensive Tablet for hypertension based on network pharmacology. China J. Chin. Mater. Med..

[84] Tang Y., Mi C., Liu J., Gao F., Long J. (2014). Compromised mitochondrial remodeling in compensatory hypertrophied myocardium of spontaneously hypertensive rat. Cardiovasc. Pathol..

[85] Van Vré E.A., Ait-Oufella H., Tedgui A., Mallat Z. (2012). Apoptotic Cell Death and Efferocytosis in Atherosclerosis. Arter. Thromb. Vasc. Biol..

[86] Xue L., Borné Y., Mattisson I.Y., Wigren M., Melander O., Ohro-Melander M., Bengtsson E., Fredrikson G.N., Nilsson J., Engström G. (2017). FADD, Caspase-3, and Caspase-8 and Incidence of Coronary Events. Arter. Thromb. Vasc. Biol..

[87] Muhammad I.F., Borné Y., Melander O., Orho-Melander M., Nilsson J., Söderholm M., Engström G. (2018). FADD (Fas-associated protein with death domain), caspase-3, and caspase-8 and incidence of ischemic stroke. Stroke.

[88] MacMahon S., Peto R., Collins R., Godwin J., Cutler J., Sorlie P., Abbott R., Neaton J., Dyer A., Stamler J. (1990). Blood pressure, stroke, and coronary heart disease *1Part 1, prolonged differences in blood pressure: Prospective observational studies corrected for the regression dilution bias. Lancet.

[89] Bai J., Yu X.J., Liu K.L., Wang F.F., Jing G.X., Li H.B., Zhang Y., Huo C.J., Li X., Gao H.L. (2017). Central administration of tert-butylhydroquinone attenuates hypertension via regulating Nrf2 signaling in the hypothalamic paraventricular nucleus of hypertensive rats. Toxicol. Appl. Pharmacol..

[90] Ungvari Z., Bagi Z., Feher A., Recchia F.A., Sonntag W.E., Pearson K., De Cabo R., Csiszar A. (2010). Resveratrol confers endothelial protection via activation of the antioxidant transcription factor Nrf2. Am. J. Physiol. Heart Circ. Physiol..

[91] Haines Z., Cull J., Baldwin S., Whitley G., Clerk A., Meijles D. (2021). BS23 mRNA Expression Profiling of Dual Specificity Phosphatases (DUSPS) in the Hypertensive Heart. BMJ J. Heart.

[92] Endo N., Toyama T., Naganuma A., Saito Y., Hwang G.-W. (2020). Hydrogen Peroxide Causes Cell Death via Increased Transcription of HOXB13 in Human Lung Epithelial A549 Cells. Toxics.

[93] Nakano R., Takahashi T., Naganuma A., Hwang G.-W. (2013). Knockdown of the gene for homeobox protein HOXB13 reduces toxicity of oxidative-stress inducers in HEK293 cells. J. Toxicol. Sci..

[94] Rodrigo R., González J., Paoletto F. (2011). The role of oxidative stress in the pathophysiology of hypertension. Hypertens. Res..

[95] Yamaguchi N., Xiao J., Narke D., Shaheen D., Lin X., Offerman E., Khodadadi-Jamayran A., Shekhar A., Choy A., Wass S.Y. (2021). Cardiac Pressure Overload Decreases ETV1 Expression in the Left Atrium, Contributing to Atrial Electrical and Structural Remodeling. Circulation.

[96] Kandoi G., Acencio M.L., Lemke N. (2015). Prediction of Druggable Proteins Using Machine Learning and Systems Biology: A Mini-Review. Front. Physiol..

[97] Bakheet T.M., Doig A.J. (2009). Properties and identification of human protein drug targets. Bioinformatics.

[98] Fauman E., Rai B.K., Huang E.S. (2011). Structure-based druggability assessment—identifying suitable targets for small molecule therapeutics. Curr. Opin. Chem. Biol..

[99] Li Z., Zhong W.-Q., Liu Z.-Q., Huang M.-H., Xie Y., Dai Z., Zou X.-Y. (2015). Large-scale identification of potential drug targets based on the topological features of human protein–protein interaction network. Anal. Chim. Acta.

[100] Kumari P., Nath A., Chaube R. (2015). Identification of human drug targets using machine-learning algorithms. Comput. Biol. Med..

[101] Li Q., Lai L. (2007). Prediction of potential drug targets based on simple sequence properties. BMC Bioinform..

[102] Costa P.R., Acencio M.L., Lemke N. (2010). A machine learning approach for genome-wide prediction of morbid and druggable human genes based on systems-level data. BMC Genom..

[103] Emig D., Ivliev A., Pustovalova O., Lancashire L., Bureeva S., Nikolsky Y., Bessarabova M. (2013). Drug Target Prediction and Repositioning Using an Integrated Network-Based Approach. PLoS ONE.

[104] Sawada R., Iwata M., Tabei Y., Yamato H., Yamanishi Y. (2018). Predicting inhibitory and activatory drug targets by chemically and genetically perturbed transcriptome signatures. Sci. Rep..

[105] Pushpakom S., Iorio F., Eyers P.A., Escott K.J., Hopper S., Wells A., Doig A., Guilliams T., Latimer J., McNamee C. (2019). Drug repurposing: Progress, challenges and recommendations. Nat. Rev. Drug Discov..

[106] Chawla N.V., Bowyer K.W., Hall L.O., Kegelmeyer W.P. (2002). SMOTE: Synthetic Minority Over-sampling Technique. J. Artif. Intell. Res..

