# Supplementary material for: Prediction of Drug Targets for Specific Diseases Leveraging Gene Perturbation Data: A Machine Learning Approach
Source: Pharmaceutics. 2022 Jan 20;14(2):234. doi: 10.3390/pharmaceutics14020234 (PMC8878225; doi:10.3390/pharmaceutics14020234)
Supplement: Supplementary file 1 [file pharmaceutics-14-00234-s001.zip › pharmaceutics-1513423-Supplementary.pdf]

pharmaceutics

Supplementary Materials: Prediction of Drug Targets for Specific Diseases Leveraging Gene Perturbation Data: A Machine Learning Approach

Kai Zhao , Yujia Shi and Hon-Cheong So

Hyperparameter tuning and weighted analysis

SVM, RF, and GBM models were implemented using "scikit-learn" in python [1], and we performed a two-step hyperparameter tuning with gridsearchCV provided in the package [2]. For SVM [3], we chose the radial basis function (RBF) as the kernel for our model, and the hyper-parameters C and gamma were chosen from (-5, 15) and (-20, 2) in log-2 space, respectively. For RF [4], we fixed the number of trees to 1000, and selected the maximum number of features (max_features) for each splitting and minimum number of samples for each leaf (min_samples_leaf) from {800, 1000, 1500, 2000, 3000, 5000} and {1, 3, 5, 10, 30, 50, 80} respectively. For GBM [5], learning rate was chosen from {0.005, 0.01, 0.015, 0.02, 0.03, 0.05}, the number of boosting iterations from the sequence from 100 to 1001 with step size 50, the maximum depth of each estimator from {2, 3, 5, 10} and maximum number of features from {10, 30, 50, 100, 500, 1000}. The subsampling proportion was fixed to 1. Finally, we implemented EN [6] using the R package "glmnet", with hyperparameter α ranging from 0 to 1 with step size 0.1 and λ following the default setting. Some refinements of the parameters grid of the above models were carried out after analyzing model fitting.

A weighted analysis was performed which increased the weight of the minority class. To illustrate this approach, for example, we have 100 observations from two classes, 10 of them positive and 90 negative. To achieve balanced class weights, we need 50 observations from the positive group and 50 observations from the negative group. The strategy is to assign class weights 50/10 and 50/90 to the positive class and negative class respectively, such that the importance of the two classes is balanced.

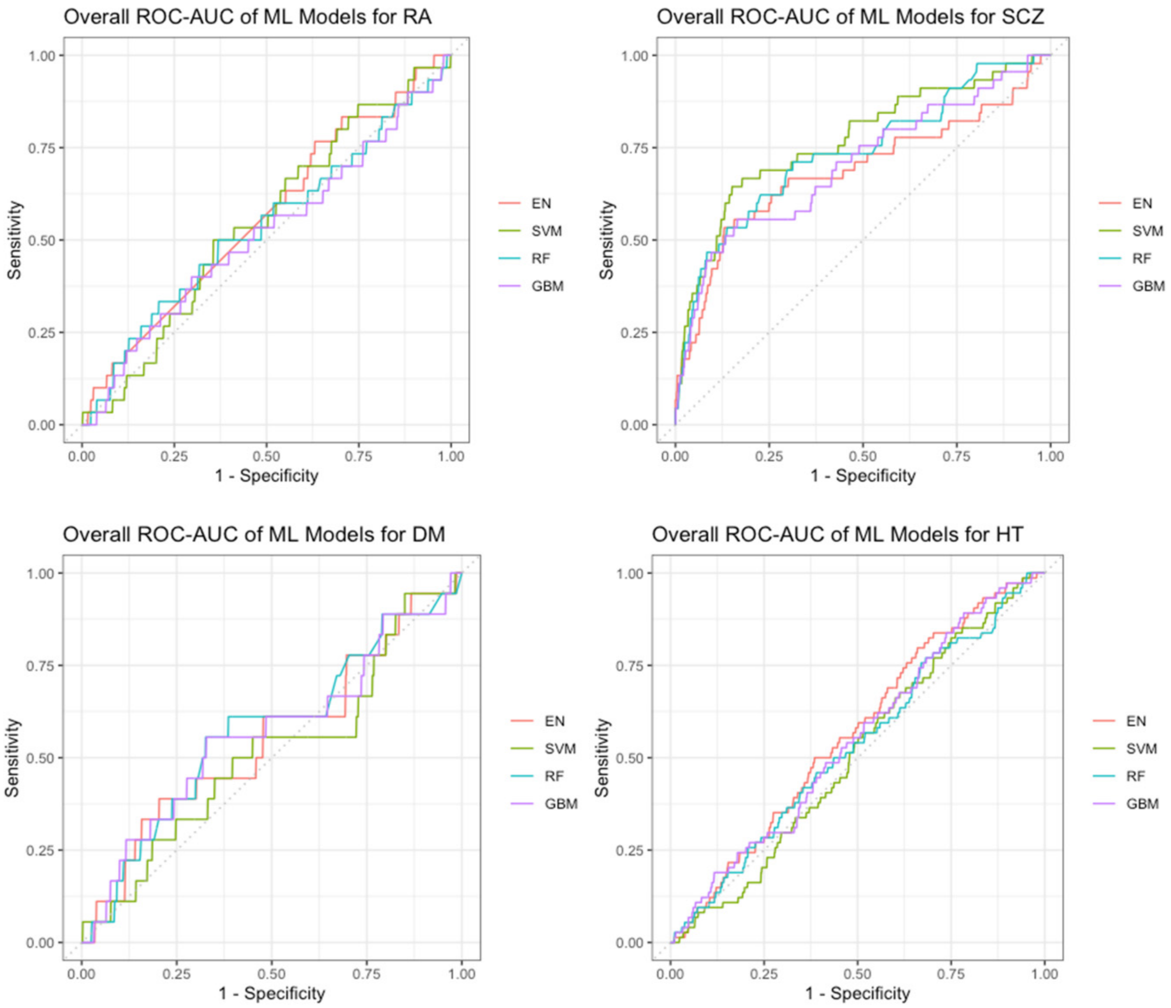

Figure S1. Receiver-operating curves (ROC) of different machine learning methods across four datasets.

This figure shows the receiver operating characteristic curve (ROC-curve) of different machine learning methods (EN, SVM, RF, GBM) across four distinct datasets, including RA, SCZ, DM and HT.

Table S1. Average predictive performance of different machine learning methods across four datasets.

	ATC DM	ATC HT	MEDI-HPS RA	ATC SCZ
		Average AUC-ROC		
SVM	0.6232	0.5433	0.5709	0.7582
RF	0.6024	0.5488	0.5706	0.7377
GBM	0.5404	0.5516	0.5244	0.7474
EN	0.6485	0.5506	0.5788	0.7496
		Average AUC-PR		
SVM	0.0834	0.0804	0.0649	0.2402
RF	0.0616	0.0884	0.0471	0.2113
GBM	0.0578	0.0937	0.0485	0.2106
EN	0.0338	0.0792	0.0706	0.2362

The figure for the best performance of learning algorithms in each dataset for different evaluation metrics is in bold. ROC-AUC: area under the curve (AUC) of the receiver operating characteristic (ROC) curve; PR-AUC: area under the curve (AUC) of the precision-recall (PR) curve. SVM: support vector machines; EN: logistic regression with elastic net regularization; RF: random forest; GBM, gradient boosted machines. MEDI-HPS: MEDication Indication - High Precision Subset; ATC: Anatomical Therapeutic Chemical classification. DM stands for diabetes mellitus, HT for hypertension, SCZ for schizophrenia, and RA for rheumatoid arthritis.

Table S2. Summary of the number of drugs in nested cross validation in our study.

Disease name	Drugs included	Avg no. of drugs in train set	Avg no. of drugs in test set
HT	74	59.2	14.8
SCZ	45	36	9
RA	30	24	6
DM	18	14.4	3.6

Table S3. Pearson correlation of predicted probabilities from different ML models for each disease.

**HT**	**SVM**	**RF**	**GBM**	**EN**
SVM	1.00	0.54	0.50	0.94
RF	0.54	1.00	0.92	0.52
GBM	0.50	0.92	1.00	0.49
EN	0.94	0.52	0.49	1.00
**DM**	SVM	RF	GBM	EN
SVM	1.00	0.37	0.38	0.41
RF	0.37	1.00	0.67	0.70
GBM	0.38	0.67	1.00	0.63
EN	0.41	0.70	0.63	1.00
**RA**	SVM	RF	GBM	EN
SVM	1.00	0.52	0.37	0.18
RF	0.52	1.00	0.79	0.47
GBM	0.37	0.79	1.00	0.41
EN	0.18	0.47	0.41	1.00
**SCZ**	SVM	RF	GBM	EN
SVM	1.00	0.57	0.52	0.59
RF	0.57	1.00	0.60	0.55

GBM	0.52	0.60	1.00	0.58
EN	0.59	0.55	0.58	1.00

Pearson correlations are shown and all are statistically significant.

Table S4. Average predictive performance of different machine learning methods across two datasets (different seed used).

	HT		RA	
	**ROC_AUC**	PR-AUC	ROC_AUC	PR-AUC
SVM	0.5355	0.0777	0.5803	0.078
RF	0.5453	0.0892	0.5412	0.0441
GBM	0.5544	0.0949	0.5367	0.0492
EN	0.5402	0.0669	0.5619	0.0339

Table S5. Enrichment test of the predicted targets for HT and RA (enrichment for targets listed in OpenTargets) [different seed used].

	HT				RA			
	SVM	RF	GBM	EN	SVM	RF	GBM	EN
1	4.33E-02	1.73E-01	3.20E-02	6.92E-03	6.09E-01	6.25E-03	1.90E-02	8.35E-02
0.8	3.17E-02	1.56E-01	2.36E-02	9.01E-03	5.26E-01	3.38E-03	9.54E-03	5.21E-02
0.6	4.61E-03	5.62E-02	1.15E-02	1.12E-03	8.40E-01	1.88E-01	6.63E-01	3.19E-01
0.4	4.99E-03	4.90E-02	9.50E-03	2.21E-03	4.98E-01	2.31E-02	1.08E-01	8.90E-02
0.2	1.63E-02	9.48E-02	2.61E-02	5.01E-04	2.63E-01	1.22E-01	1.79E-01	3.01E-01
0	8.39E-02	6.68E-01	8.34E-01	7.71E-02	9.89E-01	7.92E-01	3.64E-01	8.35E-02

Table S6 Enrichment test results from Knockdown (KD) data (5 sub-tables)

Table S6. A Enrichment test of the predicted targets from knockdown data (HT).

	Two sided p-value			
Threshold	EN	GBM	RF	SVM
1.0	5.01E-01	3.96E-01	*1.75E-02*	2.64E-01
0.8	4.50E-01	3.11E-01	*1.66E-02*	2.31E-01
0.6	7.94E-01	5.42E-01	4.41E-01	6.36E-01
0.4	8.17E-01	5.59E-01	3.90E-01	6.49E-01
0.2	7.56E-01	8.08E-01	2.79E-01	5.38E-01
0.0	6.80E-01	*3.84E-05*	8.05E-01	5.53E-01

Please also refer to the legends of main table 1. Tables S6a to 6e show how our predicted candidates were enriched for targets of HT, DM, RA, SCZ and bipolar disorder (BP) derived from the OpenTargets platform respectively. Results with p<0.05 are in italics.

Table S6. b Enrichment test of the predicted targets from knockdown data (DM).

	Two sided p-value			
Threshold	EN	GBM	RF	SVM
1.0	9.63E-01	2.77E-01	8.26E-01	6.66E-01
0.8	8.28E-01	2.60E-01	8.82E-01	6.67E-01
0.6	9.88E-01	5.74E-01	9.27E-01	5.30E-01
0.4	8.55E-01	5.48E-01	7.62E-01	6.50E-01
0.2	2.61E-01	7.77E-01	1.12E-01	4.82E-01

0.0	1.87E-01	7.45E-01	6.37E-01	2.86E-01

Results with *p*<0.05 are in italics.

Table S6. c Enrichment test of the predicted targets from knockdown data (RA).

	Two sided p-value			
Threshold	EN	GBM	RF	SVM
1.0	4.26E-01	9.54E-01	1.18E-01	7.30E-01
0.8	6.70E-01	9.23E-01	*5.16E-02*	3.00E-01
0.6	5.62E-01	9.36E-01	1.36E-01	3.06E-01
0.4	4.49E-01	5.23E-01	1.43E-01	1.31E-01
0.2	2.18E-01	2.19E-01	3.80E-01	4.91E-01
0.0	9.27E-01	1.43E-01	*4.35E-02*	6.83E-01

Results with *p*<0.05 are in italics.

Table S6. d Enrichment test of the predicted targets from knockdown data (SCZ).

	Two sided p-value			
Threshold	EN	GBM	RF	SVM
1.0	9.91E-01	6.37E-01	9.33E-01	1.17E-01
0.8	2.97E-01	9.85E-01	4.82E-01	4.71E-01
0.6	6.43E-01	2.87E-01	3.34E-01	2.64E-01
0.4	8.96E-01	2.94E-01	5.02E-01	2.42E-01
0.2	8.08E-01	6.72E-01	4.02E-01	2.95E-01
0.0	4.99E-01	4.23E-01	3.56E-01	8.85E-01

Results with *p*<0.05 are in italics.

Table S6. e Enrichment test of the predicted targets from knockdown data (Bipolar disorder).

	Two sided p-value			
Threshold	EN	GBM	RF	SVM
1.0	1.47E-01	4.55E-01	9.23E-01	2.73E-01
0.8	1.03E-01	6.77E-01	7.21E-01	3.16E-01
0.6	4.11E-01	1.98E-01	9.47E-01	1.33E-01
0.4	4.87E-01	2.42E-01	9.05E-01	1.50E-01
0.2	7.74E-01	1.88E-01	9.91E-01	2.46E-01
0.0	7.57E-01	2.41E-01	3.03E-01	3.36E-01

Results with *p*<0.05 are in italics.

Table S7a. List of identified targets (the 10 targets with the highest and lowest predicted probabilities of treatment potential are shown; four sub-tables showing targets for each disease) (Hypertension).

	SVM		RF		GBM		EN		Ensemble model	
	Gene_id	Target_name	Gene_id	Target_name	Gene_id	Target_name	Gene_id	Target_name	Gene_id	Target_name
Top 10	79862	ZNF669	498	ATP5F1A	84163	GTF2IRD2	79862	ZNF669	2104	ZNF669
	24148	PRPF6	5217	PFN2	5901	RAN	24148	PRPF6	328	ETV1
	2925	GRPR	7360	UGP2	8611	PLPP1	115201	ATG4A	2233	ATG4A
	1788	DNMT3A	5901	RAN	55278	QRSL1	2925	GRPR	720	NFE2L2
	7103	TSPAN8	8611	PLPP1	3109	HLA-DMB	6812	STXBP1	1854	RASD1

	10898	CPSF4	25793	FBXO7	55644	OSGEP	7103	TSPAN8	869	PRSS2
	6934	TCF7L2	287015	TRIM42	83942	TSSK1B	5002	SLC22A18	881	PSMB10
	8892	EIF2B2	55644	OSGEP	84124	ZNF394	6934	TCF7L2	1102	TPI1
	115201	ATG4A	3109	HLA-DMB	498	ATP5F1A	64798	DEPTOR	2172	KBTBD8
	326	AIRE	83942	TSSK1B	7639	ZNF85	6646	SOAT1	587	ITGAE
Bottom 10	3172	HNF4A	1848	DUSP6	1045	CDX2	8772	FADD	2389	ZSCAN22
	162979	ZNF296	7423	VEGFB	6626	SNRPA	58495	OVOL2	277	EBF1
	7357	UGCG	5831	PYCR1	2645	GCK	10481	HOXB13	2387	ZNF404
	51704	GPRC5B	90649	ZNF486	3488	IGFBP5	55223	TRIM62	2029	OVOL2
	51079	NDUFA13	2542	SLC37A4	9813	EFCAB14	3172	HNF4A	400	GDI1
	5029	P2RY2	4714	NDUFB8	8772	FADD	1879	EBF1	1277	FADD
	29082	CHMP4A	5720	PSME1	2101	ESRRA	4055	LTBR	172	CDKN1B
	3456	IFNB1	10247	RIDA	3788	KCNS2	2078	ERG	2183	ZNF587
	58495	OVOL2	8623	ASMTL	55163	PNPO	5867	RAB4A	623	LTBR
	1647	GADD45A	54850	FBXL12	10481	HOXB13	1027	CDKN1B	1123	UGCG

Targets highlighted in green are also discussed in the main text. In order to validate the functional relevance of our identified potential targets, we conducted a literature search of the 10 targets with the highest and lowest predicted probabilities for each disease, based on targets identified from over-expression (OE) data. As described in the introduction of main text, for targets with high predicted probabilities, we expect that up-regulation of the gene may be associated with therapeutic potential; for targets with lower-than-expected predicted probabilities, we predict that down-regulation of the gene may be associated with therapeutic potential.

Table S7. b List of identified targets for DM (the 10 targets with the highest and lowest predicted probabilities of treatment potential are shown).

	SVM		RF		GBM		EN		Ensemble model	
	Gene_id	Target_name	Gene_id	Target_name	Gene_id	Target_name	Gene_id	Target_name	Gene_id	Target_name
Top 10	1316	KLF6	25937	WWTR1	355	FAS	355	FAS	1714	WWTR1
	63933	MCUR1	355	FAS	29110	TBK1	79862	ZNF669	1128	USF1
	79862	ZNF669	79734	KCTD17	836	CASP3	23475	QPRT	2204	CREB3L1
	8431	NR0B2	7391	USF1	10898	CPSF4	7103	TSPAN8	115	KLF9
	4860	PNP	54505	DHX29	8431	NR0B2	2353	FOS	2324	FAM171B
	8859	STK19	90993	CREB3L1	9500	MAGED1	23062	GGA2	2097	KCTD17
	79960	JADE1	4942	OAT	80204	FBXO11	54505	DHX29	1228	KLF11
	8428	STK24	64170	CARD9	5518	PPP2R1A	7391	USF1	371	FOS
	3385	ICAM3	2353	FOS	10190	TXNDC9	317705	VN1R5	1045	SUOX

	25803	SPDEF	1024	CDK8	79734	KCTD17	7718	ZNF165	1451	RASGRP2
Bottom 10	10013	HDAC6	165140	OXER1	59350	RXFP1	1647	GADD45A	1409	RNF10
	6196	RPS6KA2	1591	CYP24A1	64215	DNAJC1	3172	HNF4A	879	PSMB8
	54496	PRMT7	1537	CYC1	285613	RELL2	112858	TP53RK	877	PSMB2
	6419	SETMAR	1509	CTSD	3312	HSPA8	3456	IFNB1	315	ERCC5
	2185	PTK2B	146760	RTN4RL1	7480	WNT10B	10481	HOXB13	542	IFNAR2
	112858	TP53RK	136	ADORA2B	126823	KLHDC9	2078	ERG	942	RNF5
	5582	PRKCG	126823	KLHDC9	2189	FANCG	9448	MAP4K4	856	PRKG2
	8844	KSR1	10953	TOMM34	2817	GPC1	8844	KSR1	457	H1-6
	5871	MAP4K2	10247	RIDA	6906	SERPINA7	58495	OVOL2	1769	TBK1
	9448	MAP4K4	10221	TRIB1	8521	GCM1	1879	EBF1	472	HLA-DMB

Table S7. c List of identified targets for RA (the 10 targets with the highest and lowest predicted probabilities of treatment potential are shown; four sub-tables showing targets for each disease).

	SVM		RF		GBM		EN		Ensemble model	
	Gene_id	Target_name	Gene_id	Target_name	Gene_id	Target_name	Gene_id	Target_name	Gene_id	Target_name
Top 10	55869	HDAC8	25793	FBXO7	25793	FBXO7	2115	ETV1	2025	SQOR
	23229	ARHGEF9	113878	DTX2	113878	DTX2	6522	SLC4A2	328	ETV1
	1571	CYP2E1	1622	DBI	2059	EPS8	3034	HAL	1915	SAMD4B
	121599	SPIC	55644	OSGEP	11200	CHEK2	3682	ITGAE	983	SATB1
	55847	CISD1	8771	TNFRSF6B	10769	PLK2	3164	NR4A1	1149	XBP1
	55810	FOXJ2	4092	SMAD7	1622	DBI	5198	PFAS	1061	TCF7L2
	4302	MLLT6	2059	EPS8	3326	HSP90AB1	55095	SAMD4B	869	PRSS2
	54881	TEX10	7048	TGFBR2	55278	QRSL1	7494	XBP1	1644	MCF2L
	9181	ARHGEF2	55278	QRSL1	8611	PLPP1	8703	B4GALT3	1623	SAMD4A
	64326	COP1	695	BTK	59348	ZNF350	5582	PRKCG	587	ITGAE
Bottom 10	148254	ZNF555	1026	CDKN1A	1105	CHD1	729873	TBC1D3	525	HSP90AB1
	25	ABL1	57192	MCOLN1	29994	BAZ2B	58495	OVOL2	1924	TRIM62
	166655	TRIM60	5048	PAFAH1B1	10314	LANCL1	7357	UGCG	908	RAB4A
	8495	PPFIBP2	2944	GSTM1	10270	AKAP8	1647	GADD45A	1595	NISCH
	7571	ZNF23	2108	ETFA	8915	BCL10	5867	RAB4A	1123	UGCG
	148198	ZNF98	1977	EIF4E	835	CASP2	4780	NFE2L2	247	DAXX

	8648	NCOA1	116535	MRGPRF	1977	EIF4E	3172	HNF4A	623	LTBR
	6048	RNF5	11116	FGFR1OP	11182	SLC2A6	8795	TNFRSF10B	1532	PLK2
	55635	DEPDC1	3614	IMPDH1	5696	PSMB8	4055	LTBR	1795	IL21R
	5624	PROC	165140	OXER1	5048	PAFAH1B1	1316	KLF6	1698	FBXO7

Targets highlighted in green are also discussed in the main text.

Table S7. d List of identified targets for SCZ (the 10 targets with the highest and lowest predicted probabilities of treatment potential are shown; four sub-tables showing targets for each disease).

	SVM		RF		GBM		EN		Ensemble model	
	Gene_id	Target_name	Gene_id	Target_name	Gene_id	Target_name	Gene_id	Target_name	Gene_id	Target_name
Top 10	7357	UGCG	115	ADCY9	9322	TRIP10	4907	NT5E	739	NT5E
	23475	QPRT	5089	PBX2	659	BMPR2	3725	JUN	17	ADCY9
	23609	MKRN2	2168	FABP1	3115	HLA-DPB1	9322	TRIP10	369	FLT3
	3827	KNG1	9533	POLR1C	4907	NT5E	115	ADCY9	2046	CARD9
	162963	ZNF610	167465	ZNF366	5696	PSMB8	3055	HCK	62	RHOA
	6118	RPA2	79050	NOC4L	481	ATP1B1	9697	TRAM2	94	CCND1
	23062	GGA2	85479	DNAJC5B	115	ADCY9	2212	FCGR2A	1681	MKRN2
	387	RHOA	9181	ARHGEF2	7027	TFDP1	54881	TEX10	1210	NCOA3
	29933	GPR132	56104	PCDHGB1	2538	G6PC	6505	SLC1A1	1001	SLC3A2
	64921	CASD1	205564	SENP5	117196	MRGPRX4	1812	DRD1	2151	GPR101
Bottom 10	8703	B4GALT3	6657	SOX2	5025	P2RX4	7695	ZNF136	2266	HMGB4
	2078	ERG	10013	HDAC6	2302	FOXJ1	55723	ASF1B	264	DLX6
	55662	HIF1AN	23411	SIRT1	7494	XBP1	22828	SCAF8	1579	PRSS23
	6196	RPS6KA2	55223	TRIM62	5096	PCCB	9616	RNF7	66	ARNTL
	1616	DAXX	6196	RPS6KA2	1457	CSNK2A1	6591	SNAI2	1618	SNW1
	6657	SOX2	55662	HIF1AN	5715	PSMD9	5471	PPAT	1008	SMARCE1
	6419	SETMAR	7494	XBP1	5347	PLK1	51027	BOLA1	709	NDUFS4
	84678	KDM2B	3456	IFNB1	652	BMP4	285268	ZNF621	2388	ZNF677
	55223	TRIM62	5424	POLD1	55223	TRIM62	1488	CTBP2	614	LIG1
	5424	POLD1	3458	IFNG	10629	TAF6L	808	CALM3	354	FGA

Targets highlighted in green are also discussed in the main text.

References

[1] F. Pedregosa *et al*, "Scikit-learn: Machine learning in Python," *The Journal of Machine Learning Research,* vol. 12, pp. 2825-2830, 2011.
[2] L. Buitinck *et al*, "API design for machine learning software: experiences from the scikit-learn project," *arXiv Preprint arXiv:1309.0238,* 2013.
[3] C. Cortes and V. Vapnik, "Support-vector networks," *Mach. Learning,* vol. 20, *(3),* pp. 273-297, 1995.
[4] L. Breiman, "Random forests," *Mach. Learning,* vol. 45, *(1),* pp. 5-32, 2001.
[5] J. H. Friedman, "Greedy function approximation: a gradient boosting machine," *Annals of Statistics,* pp. 1189-1232, 2001.
[6] H. Zou and T. Hastie, "Regularization and variable selection via the elastic net," *Journal of the Royal Statistical Society: Series B (Statistical Methodology),* vol. 67, *(2),* pp. 301-320, 2005.
